# Retracted: Process Evaluation of a Positive Youth Development Program in Hong Kong Based on Different Cohorts

**DOI:** 10.1155/2014/806752

**Published:** 2014-04-08

**Authors:** The Scientific World Journal

The paper titled “Process Evaluation of a Positive Youth Development Program in Hong Kong Based on Different Cohorts” [1], published in The Scientific World Journal, has been retracted as it is found to contain a substantial amount of material, without referencing, from the published article titled “Process Evaluation of a Positive Youth Development Program: Project P.A.T.H.S,” Ben M. F. Law and Daniel T. L. Shek, Research on Social Work Practice, vol. 21, no. 5, pp. 539–548, 2011.
